# PROTOCOL: Association of Antenatal Cytokine Concentrations With Neurodevelopmental Disorders of the Offspring: A Scoping Review

**DOI:** 10.1002/cl2.70053

**Published:** 2025-07-07

**Authors:** Imasha Upulini Jayasinghe, Hannah F. Jones, E. Scott Graham, Kyle Eggleton, Russell C. Dale, Jane M. Alsweiler

**Affiliations:** ^1^ Department of Paediatrics: Child and Youth Health, School of Medicine University of Auckland Auckland New Zealand; ^2^ Department of Paediatrics Neuroservices Starship Child Health, Te Toka Tumai Auckland Te Whatu Ora New Zealand; ^3^ Department of Molecular Medicine and Pathology, School of Medical Sciences University of Auckland Auckland New Zealand; ^4^ Centre for Brain Research University of Auckland Auckland New Zealand; ^5^ Department of General Practice and Primary Health Care University of Auckland Auckland New Zealand; ^6^ Kids Neuroscience Centre, The Children's Hospital at Westmead, Faculty of Medicine and Health University of Sydney Sydney New South Wales Australia

## Abstract

**Objective:**

The aim of this scoping review is to identify and map the available evidence on the association of antenatal cytokine concentrations with neurodevelopmental disorders (NDDs) of the offspring and to inform future research avenues.

**Introduction:**

NDDs are a heterogeneous group of conditions with onset in childhood which affect functioning due to altered brain development. NDDs have a multifactorial aetiology with genetic, epigenetic, and environmental factors, including the foetal intrauterine environment. Maternal immune activation (MIA) alters the intrauterine milieu of the developing foetus, and, in animal studies, has been shown to affect the brain cytoarchitecture, neuronal circuitry and glial function of the offspring. One of the mechanisms of MIA is through cytokines that may play a crucial role in affecting foetal neurodevelopment by crossing the placenta and altering cellular programming. Antenatal cytokine concentrations have the potential to be biomarkers for children at risk of NDDs, and treating a maladaptive cytokine response may reduce the risk of NDDs in these children. Therefore, it is important to understand the scope of evidence available on the association of antenatal cytokine concentration with offspring NDDs and identify the research gaps in this field.

**Inclusion Criteria:**

The review will consider all primary and grey literature which focuses on the review objective. The review will not be limited by any time period or any language of the sources of evidence.

**Method:**

The review will be conducted following the Joanna Briggs Institute (JBI) methodological framework for scoping reviews. Review questions and inclusion criteria will follow the Population, Concept, Context framework. A comprehensive search will be performed in SCOPUS, MEDLINE (Ovid), PsycINFO (Ovid), Web of Science core collection, Embase (Ovid), CINAHL databases and trial registers. Grey literature will be searched in Google Scholar, ProQuest Dissertations & Theses Global, Open Access Theses and Dissertations and library catalogues with the assistance of the librarian. Two independent reviewers will perform title‐abstract screening and full‐text screening. Eligible studies will be critically appraised using JBI critical appraisal tools. Data from eligible studies will be extracted by two reviewers using pretested data extraction tools. Findings will be presented in a final scoping review with a narrative summary and diagrammatic forms.

**Significance:**

The mapped findings and the identified research gaps will guide further research in evaluating antenatal cytokines as an early biomarker of NDDs and the potential for antenatal therapy to reduce the risk of NDDs.

**Registration:**

The review protocol is registered in the Open Science Framework (Link: https://osf.io/twsuq).

## 1 Introduction

Neurodevelopmental disorders (NDDs) are a complex, heterogeneous group of conditions with onset in childhood, which affect functioning due to altered brain development (Morris‐Rosendahl and Crocq 2020; Parenti et al. 2020; Srivastava et al. 2019). NDDs include autism spectrum disorder (ASD), attention‐deficit/hyperactivity disorder (ADHD), intellectual disability, communication disorders, specific learning disorders, and neurodevelopmental motor disorders such as Tourette syndrome (American Psychiatric Association 2013). NDDs were initially considered to be limited to childhood (Thapar et al. 2017) but recent observational studies have shown that NDDs often persist across the lifespan and impact quality of life and function in adulthood (Antolini and Colizzi 2023). In addition, NDDs often coexist with other NDDs or neuropsychiatric disorders such as depression (Gaudet and Gallagher 2020; Parenti et al. 2020; Thapar et al. 2017). There is a significant prevalence of NDDs, such as ASD and ADHD, in both high‐income and low‐middle‐income countries (Bitta et al. 2017; Francés et al. 2022, 2023; Kang et al. 2023; Yang et al. 2022). Current trends show a rising prevalence of NDDs across the world (Chiarotti and Venerosi 2020; Kang et al. 2023; Zablotsky et al. 2019). Despite their high frequency, diagnosing NDDs is challenging because of their multifactorial aetiology, variability in clinical presentation, and different NDD classification systems (Cainelli and Bisiacchi 2023; Gaudet and Gallagher 2020; McPartland 2016; Thapar et al. 2017).

Multiple factors, including genetic, epigenetic, and environmental factors, contribute to the multifactorial origin of NDDs (De Felice et al. 2015; Morris‐Rosendahl and Crocq 2020). Significant heritability is evident in many NDDs, such as ASD and ADHD (Gidziela et al. 2023). However, the genetic architecture of NDDs is complex, and the expression of genetic vulnerability can be influenced by epigenetic and environmental factors (Banik et al. 2017). Therefore, the intrauterine environment of the foetus, influenced by maternal factors, can potentially affect the offspring's neurodevelopment significantly (De Felice et al. 2015; Han, Patel, Jones, and Dale 2021; Han, Patel, Jones, et al. 2021; Solek et al. 2018). Maternal immune activation (MIA) animal models demonstrate that foetal exposure to an altered maternal immunological milieu can affect the normal neurodevelopmental processes of the foetus (Bergdolt and Dunaevsky 2019; Han, Patel, Jones, et al. 2021). Findings from preclinical animal models show that MIA affects the normal neurodevelopment of the offspring, bringing changes in the brain cytoarchitecture (Gumusoglu et al. 2020; Loayza et al. 2023), neuronal circuitry (Kalish et al. 2021), and glial function (Loayza et al. 2023; Ozaki et al. 2020). Even though the underlying mechanisms are unclear, clinical observations show that both chronic and acute inflammatory conditions of the mother during pregnancy, are associated with NDDs of the offspring (Han, Patel, Jones, and Dale 2021; Han, Patel, Jones, et al. 2021; Jiang et al. 2016).

One of the mechanisms driving the immune pathways of MIA is the immune signalling molecules, cytokines, and chemokines, which can cross the placenta and foetal blood–brain barrier to affect foetal neurodevelopment (Han, Patel, Jones, and Dale 2021; Han, Patel, Jones, et al. 2021). Animal models demonstrate that the pro‐inflammatory cytokines IL‐6 and IL‐17A are key mediators of neurodevelopmental effects in the offspring (Choi et al. 2016; Smith et al. 2007; Wu et al. 2017), and early translational work suggests that elevated concentrations of IL‐6 and IL‐17A are also associated with NDDs in humans (Boulanger‐Bertolus et al. 2018; Fujitani et al. 2022; Goines and Ashwood 2013; Graham et al. 2018; Rudolph et al. 2018; Wong and Hoeffer 2018). Other cytokines such as TNF, IL‐13, IL‐1β, IL‐2 and IL‐4 have also been associated with NDDs (Goines and Ashwood 2013; Han, Patel, Jones, et al. 2021; Ponzio et al. 2007; Zengeler and Lukens 2021). Studies from animal models have shown this relationship may be causal, with abnormal neural development and differentiation in mice exposed to cytokines, resulting in changes in their behavioural patterns (Choi et al. 2016; Fujitani et al. 2022; Gumusoglu et al. 2020; Kalish et al. 2021; Loayza et al. 2023; Ozaki et al. 2020; Smith et al. 2007; Wu et al. 2017; Zengeler and Lukens 2021). In humans, several population birth cohort studies have shown that maternal inflammatory proteins, including cytokines, may be associated with the neurodevelopment of their offspring (Abdallah et al. 2013; Gilman et al. 2017; Goines et al. 2011). However, evidence on the association of cytokines with the causation of NDDs in humans is still emerging.

At present, the diagnosis of NDDs is mainly by subjective evaluation of behaviour and clinical judgement based on different diagnostic criteria such as the DSM‐5 (American Psychiatric Association 2013; Gaudet and Gallagher 2020). Molecular diagnoses are increasingly possible using advanced genetic testing and functional analysis (Parenti et al. 2020), but for NDDs, which also carry epigenetic and environmentally driven causation, there is a need to identify biomarkers which segregate with NDD subtypes and have the potential to direct treatment in the future (McPartland 2016). Antenatal cytokine concentrations may be a promising biomarker for early identification and classification of NDDs, and treating a maladaptive cytokine response may reduce the risk of NDDs. Therefore, it is important to understand the scope of evidence available on the association of antenatal cytokines and offspring NDDs in the literature and to explore areas where more research is needed. A comprehensive preliminary search done on 25th of September 2023 in PubMed, Medline (Ovid), Cochrane Library, PROSPERO, and Joanna Briggs Institute (JBI) evidence synthesis sources did not reveal any similar earlier or registered systematic or scoping reviews on this study question. This recognises the necessity of an evidence synthesis on the role of antenatal cytokines in NDDs of the offspring. To address this, a scoping review was identified as the most suitable since the evidence synthesis is not intended to inform the development of clinical guidelines or clinical practice, but to explore the available evidence in the literature. This scoping review aims to identify and map the evidence on the association of antenatal cytokine profiles with NDDs of the offspring in terms of the cytokines assessed, their association with NDDs, gestational period at which antenatal cytokine concentration was assessed, type of sample used for cytokine assessment, offspring age at neurodevelopmental assessment, measures or tools used for neurodevelopmental assessment of the offspring and, to inform the avenues for future research in this field.

## 2 Review Question/s

What evidence is available on the association of antenatal cytokine concentrations with NDDs of the offspring?

### 2.1 Sub Questions

1. What cytokines have been assessed during pregnancy, at which gestational period, on what type of sample, and using what methods of measurement?
2. What was the age of offspring at neurodevelopmental assessment, and what measures or tools were used for their assessment?
3. What are the reported associations of specific antenatal cytokines with specific NDDs of the offspring, and how are they measured and reported?

## 3 Eligibility Criteria

### 3.1 Population

This review will consider all studies with pregnant women as their study population. Pregnant mothers could be of any age and at any gestational age when the biochemical assessment for cytokine concentration was done. As the scoping review aims to capture a wide range of evidence from the literature, no exclusions will be made based on maternal comorbidities.

### 3.2 Concept

The concept under consideration for this review is the association of antenatal cytokine concentrations with the NDDs of their offspring. As the body of evidence on human studies to show any association between antenatal cytokines and offspring NDDs is still emerging, this review will include all human studies that describe any of the cytokine/s concentrations assessed at least once during the mother's antenatal period and/or at delivery on any of the samples (serum/plasma/amniotic fluid), and its association with any of the NDD assessed using any measure or tool, at any age of the offspring. To have a broader range of evidence sources, this review will also include sources which have studied neurodevelopmental outcomes, such as executive functioning (without a definitive diagnosis as an NDD), in association with antenatal cytokine concentration. The exclusion criteria are animal studies and human studies in which cytokine assessment is confined only to the postnatal period.

### 3.3 Context

As the scoping review aims to have a broad evidence base, the review will include studies from any contextual setting. Evidence from any geographical location and any socioeconomic background will be eligible for the review.

## 4 Types of Resources

This review will include all types of primary studies irrespective of the methodology, including descriptive studies (case reports, case series), analytical observational studies (cross‐sectional studies, ecological studies, cohort studies and case‐control studies) and experimental studies (randomised controlled trials, quasi‐experimental studies, and non‐randomised controlled trials). The review criteria will include all published original research articles and short reports. This review will also include eligible sources from unpublished (grey) literature, accessed from library databases, research repositories, conference proceedings, theses/dissertations repositories, and preprints. The review will not include published protocols, commentaries, communications, perspectives, editorials, and letters to the editor. Evidence synthesis literature, including systematic reviews, meta‐analyses, evidence gap maps, scoping reviews and narrative reviews, will not be included in the final review to prevent data duplication. However, these evidence syntheses sources will be screened to identify other eligible primary sources from their references that have not been included in the review from the primary search.

## 5 Methodology

The scoping review will follow the JBI methodology 2020 updated guidance (Munn et al. 2022; Peters et al. 2015, 2020; Pollock et al. 2021). The review will also utilise and align with the Preferred Reporting Items for Systematic Reviews and Meta‐Analyses extension for scoping reviews (PRISMA‐ScR) reporting guideline and checklist (Tricco et al. 2018). (The protocol for this scoping review is registered in the Open Science Framework. Link: https://osf.io/twsuq)

## 6 Search Strategy

In compliance with the JBI methodology, this review will use a three‐step search strategy (Aromataris and Mann 2020; Peters et al. 2020; Pollock et al. 2021), and follow the Searching for studies guide for Campbell systematic reviews (MacDonald et al. 2024). Based on the population, concept, and context (PCC) elements of the research question, the search string of this review will include synonyms for the search terms (pregnancy, cytokine profile and NDDs), which are combined with OR and AND Boolean operators. During the review protocol development, the first step of the search strategy, which is the limited basic search of the key terms in at least two online databases, was completed. This included a preliminary search in MEDLINE (Ovid) and Scopus online databases (Supporting Information S1: Appendix 1). Titles and abstracts of the retrieved articles from this basic search were analysed to identify other relevant keywords and the index terms used to describe the articles. These keywords and index terms were used to develop the full search string. The full search string for the MEDLINE (Ovid) database is provided in Supporting Information S2: Appendix 2.

The second step will include a detailed second search using all the related keywords and index terms identified across all the included databases. The databases searched will be SCOPUS, MEDLINE (Ovid), PsycINFO (Ovid), Web of Science core collection, Embase (Ovid), CINAHL and the clinical trials registers including CENTRAL, clinicaltrials.gov and WHO ICTRP. If additional keywords and potentially useful search terms are discovered during these searches, they will be incorporated into the search string. The same search string will be used across all the databases, with only database‐specific changes. Medical Subject Headings (MeSH terms) will be mapped and used as specific to the databases which use subject headings. Online database searches will not be limited by any time period, any language, or any type of evidence sources.

The third step will include searching the reference list of articles to find additional sources. ‘Citation Chaser’ (Link: https://estech.shinyapps.io/citationchaser/) will be used as forward and backward citation chasing of all selected articles after title and abstract screening, to find additional relevant studies not retrieved by the database searches (Haddaway et al. 2022). Targeted hand‐searching of the included full‐text articles after the full‐text screening will be done to find any additional sources for the review. Even though the final review will not include evidence syntheses sources such as systematic reviews, their reference lists will be hand‐searched to identify additional eligible primary studies for the review.

During the conceptual development of the review, the university library research services advisor of the Faculty of Medicine and Health Sciences, University of Auckland, was consulted to decide on suitable databases and other evidence sources for the review. Assistance was also obtained to develop and refine the final search strategy for the online databases. Further assistance will be obtained during the database and grey literature searches. Grey literature will be searched in Google Scholar, ProQuest Dissertations & Theses Global, Open Access Theses and Dissertations and in library catalogues with the assistance of the librarian. To capture a wider range of grey literature, the Google Scholar search will not be limited by the number of pages but will include all results from the initial search to the title‐abstract screening of the review (Haddaway et al. 2015). Authors or relevant organisations will be contacted if necessary to obtain further evidence for the review.

## 7 Study Selection

Following the search of databases and grey literature, the retrieved sources will be uploaded into the Rayyan web‐based tool (Link: https://www.rayyan.ai/) for de‐duplication, screening, and selection of the sources. Based on the predefined inclusion criteria, the sources will undergo initial screening by reviewing the title and abstract for keywords/terms of the review. Potentially relevant sources will then be retrieved and examined in more detail by full‐text screening to decide whether they fit the review's inclusion criteria. Sources of evidence that follow all inclusion criteria based on PCC elements will be included for the final review. Screening and selection of the sources for the final review will be conducted independently by two reviewers (I. U. J. and H. F. J.). Before the initial title‐abstract screening for study selection, the process will be pilot‐tested in three databases (SCOPUS, EMBASE, MEDLINE). The first 25 articles retrieved from the final search strategy in each database will be pilot‐tested for the title‐abstract screening independently by two reviewers involved in the screening and selection process (I. U. J. and H. F. J.), and a third reviewer (J. M. A.) will be consulted if any discrepancies occur. Once all reviewers are in consensus, the title‐abstract screening and full text screening of the sources will be conducted by the two dedicated reviewers, I. U. J. and H. F. J., independently in all databases. Selected sources by each reviewer will be cross‐checked, and eligible sources will be finalised and included in the review. Any disagreements during the process will be managed through discussion between the two reviewers. Any further uncertainties will be consulted through a third reviewer, JA. Targeted hand searching of the reference lists of included full‐text articles and the evidence syntheses sources will be done by a single reviewer (I. U. J.), and the identified articles will be finalised following discussion with the review team.

The selection process of sources will be mapped using the adapted PRISMA 2020 flow diagram to show the inclusion and exclusion process of the sources. Reasons for the exclusion of full‐text articles after title‐abstract screening will be reported with reasons in an appendix of the scoping review report.

Because of the broad nature of the scoping review, if new potentially relevant terms, concepts, and evidence sources arise during the searching, screening and selection process, the search may be modified and expanded to account for those eventualities. Any changes or modifications done to this protocol will be clarified in the final scoping review report.

## 8 Assessment of Methodological Quality

While it is not mandatory to assess the methodological quality or risk of bias in individual sources for a scoping review (Aromataris and Mann 2020; Peters et al. 2020, 2022; Pollock et al. 2021, 2023), the authors aim to inform future avenues for research in the field of antenatal cytokine concentrations and offspring NDDs. Therefore, to have a structured, scientific, and critically examined base for the available evidence, this scoping review will include an assessment of the methodological quality of included sources of evidence for the final review. Critical appraisal of the methodological quality of the included sources will be assessed using JBI's standardised tools specific to the study designs of the primary source (Joanna Briggs Institute n.d.). Critical appraisal will not be done for grey literature evidence sources if a specific study design cannot be identified as compatible with the JBI's standardised critical appraisal tools. The methodological quality assessment results will be reported in a narrative summary in the results section and as a supporting file of the scoping review report. The strengths and limitations of each source of evidence will also be reported. A summary statement on the overall quality of available evidence will be stated with recommendations for future research. This scoping review will not include a synthesis of evidence from the results in primary sources of evidence. Also, the methodological quality assessment of sources will not influence the inclusion and exclusion of sources in the final review.

## 9 Data Extraction

Data extraction for the scoping review will focus on charting data in compliance with the review objective and its PCC elements. This will include variables related to details of the evidence source, maternal gestation, biochemical assessment of cytokines, neurodevelopmental assessment of offspring and its outcomes and the associations reported between antenatal cytokines and offspring NDDs. Specific details extracted from the evidence source will include the type of source, Doi, first author, published year, participants' country of origin, study purpose/aim, study design, sample size, reported strengths and limitations by the authors. Specific antenatal cytokine concentration assessment details will include mean age of study participants, characteristics of the participants (e.g., any excluded comorbidities), gestational period at which the sample collected for cytokine assessment, type of sample in which cytokine concentration assessed (plasma/serum/amniotic fluid), time of the sample collection, reported value/concentrations of specific cytokines assessed, use of controls or comparison group for normative data, reference ranges/detection ranges used, method used in cytokine measurements, sensitivity of the measurement method, and duration from sample collection to sample assessment. Offspring neurodevelopmental assessment details will include age at assessment, measures or tools used, NDDs reported, reported association between cytokine and NDD, and the method of determining this association. The draft data extraction table (Supporting Information S3: Appendix 3) will be created on MS Excel and will also accompany a data extraction guidance form (Supporting Information S3: Appendix 3) for the table. To ensure the comprehensiveness of the data extraction table, the draft table will be pilot‐tested by two reviewers (I. U. J. and H. F. J.) on at least two sources from each type of evidence source included for the review. After pilot testing, the review group will have a meeting to refine and update the data extraction form. The final data extraction form and the guidance sheet used will be included in the scoping review report. Two reviewers (I. U. J. and H. F. J.) will independently extract data from all the sources included in the final review. Any disagreements will be resolved by discussion between the reviewers. Data extracted will be recorded separately in MS Excel data sheets by each reviewer (I. U. J. and H. F. J.). The final data set for review will be decided by discussion between all members of the review team and created in a separate data sheet in MS Excel.

## 10 Data Analysis and Presentation

Analysis of extracted data will align with the review objective and questions. IUJ will analyse the final data set from data extraction. Data will be summarised to show the different associations, based on cytokine and the NDD reported. Results pertaining to available evidence in this field will be mapped based on conceptual categories including gestational age, offspring age, type of NDD reported, type of measure/tool used in offspring assessment, type of cytokine assessed, type of sample used, time of sample collection, method of cytokine measurement and reporting, use of controls or comparison group and method in describing the association of cytokine with NDD. Quantitative analysis of results will include descriptive statistics reported as frequency, proportion, or percentages. Data will be presented in tabular form, accompanied by a narrative description to present further characteristics and details of the variables. Additionally, data will be presented using visual presentations such as pie charts, bubble maps, tree graphs, word clouds, and so forth. All visual presentations will accompany a narrative summary of the results. Results of quality assessment in included studies will be presented in a supporting file of the review. The scoping review will note the research gaps in this field and discuss them in detail.

The final scoping review report will include a completed PRISMA‐ScR checklist (Tricco et al. 2018). Final conclusions of the scoping review will be drawn from the mapped evidence and a summary will be included.

## 11 Significance of the Review

The review will examine the breadth of evidence that antenatal cytokine concentrations in pregnancy are associated with NDDs in the offspring, and map and summarise the available evidence. The review will elaborate on the research gaps identified through the scoping review and provide directions for future research. The review findings may guide further research in evaluating cytokines during pregnancy as early biomarkers of NDDs and their potential role in the pathophysiology and therapeutics of NDDs. Therefore, the scoping review results have the potential to contribute to both the clinical care and the understanding of the epidemiology of NDDs.

## Author Contributions

Imasha Upulini Jayasinghe developed the review question, conducted the literature review, designed the methodology of the review, drafted the original protocol, and conducted the manuscript revisions. Hannah F. Jones and Jane M. Alsweiler contributed to conceptualising, designing, and critically revising the manuscript. Kyle Eggleton, E. Scott Graham, and Russell C. Dale assisted with designing the review and critically revising the manuscript. All authors approved the final protocol for the review.

## Conflicts of Interest

The authors declare no conflicts of interest.

## Supporting information

APPENDIX_1_SuppInfo.pdf.

APPENDIX_2_SuppInfo.pdf.

APPENDIX_3_SuppInfo.pdf.
