# Supplementary material for: PROTOCOL: Association of Antenatal Cytokine Concentrations With Neurodevelopmental Disorders of the Offspring: A Scoping Review
Source: Campbell Syst Rev. 2025 Jul 7;21(3):e70053. doi: 10.1002/cl2.70053 (PMC12230768; doi:10.1002/cl2.70053)
Supplement: Supplementary file 1 — APPENDIX_1_SuppInfo.pdf. [file CL2-21-e70053-s003.pdf]

APPENDIX 1

Preliminary search strategy

a) ***Search string with the number of sources for MEDLINE (Ovid)***

(Search done on 11.10.2023. The search was mapped with subject headings.)

SEARCH STRING

Ovid MEDLINE(R) Epub Ahead of Print, In Process & Other Non-Indexed Citations, Ovid MEDLINE (R) Daily, and Ovid MEDLINE (R) 1946-Present

1 Pregnancy/ 994735

2 Pregnant Women/ 14850

3 Obstetrics/ 24744

4 Mothers/ 56631

5 Prenatal Diagnosis/ 40491

6 (Pregnan* or "Pregnant wom?n" or "Pregnant mother*" or Maternal or Mother* or Gestation* or Obstetric* or Prenatal or Perinatal).mp. [mp=title, book title, abstract, original title, name of substance word, subject heading word, floating sub-heading word, keyword heading word, organism supplementary concept word, protocol supplementary concept word, rare disease supplementary concept word, unique identifier, synonyms, population supplementary concept word, anatomy supplementary concept word] 1526281

7 1 or 2 or 3 or 4 or 5 or 6 1526281

8 Cytokines/ 179604

9 Chemokines/ 17903

10 Interferons/ 26101

11 Interleukins/ 18821

12 (Cytokine* or "Cytokine profile*" or Chemokine* or Interferon or Interleukin).mp. [mp=title, book title, abstract, original title, name of substance word, subject heading word, floating sub-heading word, keyword heading word, organism supplementary concept word, protocol supplementary concept word, rare disease supplementary concept word, unique identifier, synonyms, population supplementary concept word, anatomy supplementary concept word] 907423

13 8 or 9 or 10 or 11 or 12 913871

14 7 and 13 27404

15 Child Development/ or Developmental Disabilities/ 70417

16 Autistic Disorder/ or Neurodevelopmental Disorders/ or Attention Deficit Disorder with Hyperactivity/ 64041

17 Autistic Disorder/ or Autism Spectrum Disorder/ or Child Development Disorders, Pervasive/ 47754

18 Autism Spectrum Disorder/ or Asperger Syndrome/ 21858

19 Attention Deficit Disorder with Hyperactivity/ 35008

20 Tourette Syndrome/ 4811

21 Tic Disorders/ or Tourette Syndrome/ or Tics/ 6458

22 Learning Disabilities/ 14678

23 Autistic Disorder/ 25688

24 ("Neurodevelopmental outcome*" or "Neurodevelopmental disorder* " or Autism or ASD or "autism spectrum disorder*" or "Autism-spectrum disorder* " or Autistic or ADHD or "Attention deficit hyperactive disorder* " or "Attention-deficit-hyperactive disorder* " or "Attention deficit hyperactiv* " or Hyperactiv* or TS or "Tourette syndrome" or "Tics disorder 0R Learning disability" or LD).mp. [mp=title, book title, abstract, original title, name of substance word, subject heading word, floating sub-heading word, keyword heading

word, organism supplementary concept word, protocol supplementary concept word, rare disease supplementary concept word, unique identifier, synonyms, population supplementary concept word, anatomy supplementary concept word] 240334

25 15 or 16 or 17 or 18 or 19 or 20 or 21 or 22 or 23 or 24 316063

26 14 and 25 800

b. <u>Search string with the number of sources for SCOPUS.</u>

(Search done on 11.10.2023)

<u>SEARCH STRING</u>

(TITLE-ABS-KEY (cytokine* OR "cytokine profile*" OR chemokine* OR interferon OR interleukin)) AND (TITLE-ABS-KEY ((pregnan* OR "pregnant wom?n" OR "pregnant mother*" OR maternal OR mother* OR gestation* OR obstetric* OR prenatal OR perinatal))) AND (TITLE-ABS-KEY (("neurodevelopmental outcome*" OR "neurodevelopmental disorder* " OR autism OR asd OR "autism spectrum disorder*" OR "autism-spectrum disorder* " OR autistic OR adhd OR "attention deficit hyperactive disorder* " OR "attention-deficit-hyperactive disorder* " OR "attention deficit hyperactiv* " OR hyperactiv* OR “ts” OR "tourette syndrome" OR "tics disorder or learning disability" OR ld)))

Number of results - 1,233 (without any filters)
