# Supplementary material for: PROTOCOL: Association of Antenatal Cytokine Concentrations With Neurodevelopmental Disorders of the Offspring: A Scoping Review
Source: Campbell Syst Rev. 2025 Jul 7;21(3):e70053. doi: 10.1002/cl2.70053 (PMC12230768; doi:10.1002/cl2.70053)
Supplement: Supplementary file 2 — APPENDIX_2_SuppInfo.pdf. [file CL2-21-e70053-s002.pdf]

APPENDIX 2

Full Search Strategy for MEDLINE (Ovid)

(Search done on 27.03.2025. The search was mapped with subject headings, and no limits were applied.)

SEARCH STRING

Ovid MEDLINE(R) Epub Ahead of Print, In Process & Other Non-Indexed Citations, Ovid MEDLINE (R) Daily, and Ovid MEDLINE (R) 1946-Present

1 Pregnancy/ 1036445

2 Pregnant Women/ 15994

3 Maternal Health/ 2742

4 Mothers/ 60890

5 Obstetrics/ 25462

6 Prenatal Exposure Delayed Effects/ or Prenatal Diagnosis/ 77950

7 (Pregnan* or "Pregnant wom#n" or "Pregnant mother*" or Matern* or Mother* or "mother-to-be" or "mothers-to-be" or (expectant* adj2 mother*) or expectant* or "before delivery" or childbearing or child-bearing or gravidit* or "before delivery" or antenatal* or ante-natal* or antepartum or ante-partum or Gestation* or Obstetric* or Prenatal* or pre-natal* or Perinatal* or peri-natal*).mp. [mp=title, book title, abstract, original title, name of substance word, subject heading word, floating sub-heading word, keyword heading word, organism supplementary concept word, protocol supplementary concept word, rare disease supplementary concept word, unique identifier, synonyms, population supplementary concept word, anatomy supplementary concept word] 1641216

8 Cytokines/ 190750

9 Chemokines/ 18507

10 Interferons/ 26769

11 Interleukins/ 19559

12 Tumor Necrosis Factor- alpha/ 0

13 (Cytokine* or "Cytokine profile*" or Chemokine* or Interferon* or Interleukin* or "tumo?r necrosis factor" or IL or TNF or IFN).mp. [mp=title, book title, abstract, original title, name of substance word, subject heading word, floating sub-heading word, keyword heading word, organism supplementary concept word, protocol supplementary concept word, rare disease supplementary concept word, unique identifier, synonyms, population supplementary concept word, anatomy supplementary concept word] 1217432

14 Child Development/ 52741

15 exp Neurodevelopmental Disorders/ 223242

16 Autism Spectrum Disorder/ 23771

17 Autistic Disorder/ 28040

18 Child Development Disorders, Pervasive/ 6703

19 Asperger Syndrome/ 1801

20 Attention Deficit Disorder with Hyperactivity/ 37307

21 Language Disorders/ 6807

22 Language Development Disorders/ 7708

23 Communication Disorders/ 3097

24 Tourette Syndrome/ 4986

25 Tics/ 1296

26 Tic Disorders/ 1955

27 Stereotypic Movement Disorder/ 636

28 Motor Skills Disorders/ 3378

29 Mutism/ 1195

30 Learning Disabilities/ 14845

31 Dyslexia/ 8745

32 Intellectual Disability/62239

33 Developmental Disabilities/ 23440

34 ("Neurodevelopment* outcome*" or "Neurodevelopment* disorder*" or ASD or Autism or "Autism spectrum disorder" or "Autism-spectrum disorder" or Autistic or "Asperger* syndrome" or Asperger* or ADHD or "attention deficit disorder*" or "Attention deficit hyperactiv* disorder" or "Attention-deficit-hyperactiv* disorder" or "Attention deficit hyperactiv*" or Hyperactiv* or TS or "Tourette* syndrome" or Tourette* or "Tics disorder" or Tics or "stereotypic movement disorder*" or "motor skill* disorder*" or mutism or "Learning disabilit*" or LD or "Learning difficult*" or "Intellectual disabilit*" or "intellectual development* disorder*" or "Development* delay*" or "language disorder*" or "language development* disorder*" or "communication disorder*" or "Global development* delay*" or "Development* disabilit*" or "Child development* disorder*" or "Child development").mp. [mp=title, book title, abstract, original title, name of substance word, subject heading word, floating sub-heading word, keyword heading word, organism supplementary concept word, protocol supplementary concept word, rare disease supplementary concept word, unique identifier, synonyms, population supplementary concept word, anatomy supplementary concept word] 455462

35 1 or 2 or 3 or 4 or 5 or 6 or 7 1641216

36 8 or 9 or 10 or 11 or 12 or 13 1217432

37 14 or 15 or 16 or 17 or 18 or 19 or 20 or 21 or 22 or 23 or 24 or 25 or 26 or 27 or 28 or 29 or 30 or 31 or 32 or 33 or 34 483577

38 35 and 36 and 37 1196
