# Supplementary material for: PROTOCOL: Association of Antenatal Cytokine Concentrations With Neurodevelopmental Disorders of the Offspring: A Scoping Review
Source: Campbell Syst Rev. 2025 Jul 7;21(3):e70053. doi: 10.1002/cl2.70053 (PMC12230768; doi:10.1002/cl2.70053)
Supplement: Supplementary file 3 — APPENDIX_3_SuppInfo.pdf. [file CL2-21-e70053-s001.pdf]

APPENDIX 3

Data extraction table

1	Type of evidence source	
2	Topic	
3	DOI	
4	Place of publication	
5	First author	
6	Year and month of publication	
7	Study design	
8	Study purpose/aim	
9	Country	
10	Sample size	
11	Age of mothers	
12	Characteristics of the sample	
13	Gestational age at cytokine assessment	
14	Cytokine assessment method	
15	Sensitivity of the assessment method	
16	Type of sample used to measure cytokine concentration	
17	Time of the day when sample was collected	
18	Time duration from sample collection to sample analysis	

19	Sample storing details	
20	Type/s of cytokine assessed	
21	Reported value/concentration of the cytokine and whether high, low or normal	
22	Use of controls or comparison group for normative data	
23	Detection range/s of the measured cytokines (for each cytokine)	
24	Offspring's age at assessment	
25	Measures/ tools used in offspring assessment	
26	Specific NDD/NDO studied/reported	
27	Association reported between specific cytokine level and specific NDD/NDO	
28	Method used to determine association	
29	Reported confounders for associations	
30	Reported strengths by authors	
31	Reported limitations by authors	

Data extraction guidance form

1	Type of evidence source	Mention the type of the source (ex: research article, short paper, thesis, conference proceeding)
2	Topic	Full title of the source
3	DOI / link	DOI or link to the source

4	Place of publication	Where is the source available? (ex: journal name, library name, conference name, organisation name)
5	First author	Name of the first author as mentioned in the source
6	Year and month of publication	Year and month of publication
7	Study design	Study design as mentioned in the methodology (ex: prospective cohort, retrospective cohort, case control)
8	Study purpose/aim	Purpose or aim of the study as mentioned in the article
9	Country	Country of participants in whom the study was conducted
10	Sample size	Final sample size included for the study
11	Age of mothers	Mean (SD) age of the mothers
12	Characteristics of the sample	Any comorbidities excluded/included.<br>Any other special characteristics of the study sample
13	Gestational age at cytokine assessment	Gestational age in weeks (mean with SD or median with IQR) at which the cytokine assessment is done. If several assessments are done, mention the gestational age at each instance separately.
14	Cytokine assessment method	Method used in biochemical assessment of the plasma cytokine level.<br><br>If several methods are used, clarify and separately mention tests for which cytokine and at which gestational age.
15	Sensitivity of the assessment method	Sensitivity of the cytokine assessment method as mentioned in the article

16	Type of sample used to measure cytokine concentration	Type of the sample in which the cytokine concentration was measured.
17	Time of the day when sample was collected	Time of the day when the samples were collected for cytokine measurement.
18	Time duration from sample collection to sample analysis	Time gap from sample collection to sample measurement (in years)
19	Sample storing details	How the sample was stored. (mention the temperatures at which the samples were stored and specific sample processing details if provided)
20	Type/s of cytokine assessed	Mention all the cytokines assessed.<br>If repeated assessments are done, mention separately which cytokines assess at which GA of the mother.
21	Reported value/concentration of the cytokine and whether high, low or normal	For each cytokine assessed, mention the mean or median (or any value with the type of value) concentration value reported. Also mention whether this is high, normal or low level with the reference ranges as reported in the study Mention details if these were compared between groups (ex: cases and controls) and values observed in each group.<br><br>For repeated assessments, mention separately for each cytokine at each GA.
22	Use of controls or comparison group for normative data	Mention whether a comparison group was used for normative data with their characteristics.<br>Mention the reference ranges used for reporting the cytokine measurement.

23	Detection range/s of the measured cytokines (for each cytokine)	Mention the detection ranges (LLOD and/or ULOD) for each cytokine measured in the article.
24	Offspring's age at assessment	Age at which assessment done in the offspring
25	Measures/ tools used in offspring assessment	Specific measures or tools used for the assessment of the offspring. Mention the full name of the assessment (not the short forms).
26	Specific NDD/NDO studied/reported	Reported NDDs/NDOs in the study
27	Association reported between specific cytokine level and specific NDD/NDO	Observed association between cytokine and NDD/NDO.<br>Mention separately for each cytokine and each NDD/NDO. If repeated assessments are done, mention the reported associations at each assessment.
28	Method used to determine association	The method of determining the association between the variables (ex: correlation). Mention separately for all the associations reported.
29	Reported confounders for associations	Reported confounders or allowance for confounding effect in each association reported.
30	Reported strengths by authors	Strengths of the study as reported by the authors (in point form)
31	Reported limitations by authors	Limitations of the study as reported by the authors (in point form)
